# Posterior Summarization in Bayesian Phylogenetics Using Tracer 1.7

**DOI:** 10.1093/sysbio/syy032

**Published:** 2018-04-27

**Authors:** Andrew Rambaut, Alexei J Drummond, Dong Xie, Guy Baele, Marc A Suchard

**Affiliations:** 1 Institute of Evolutionary Biology, University of Edinburgh, Ashworth Laboratories, King’s Buildings, Edinburgh, EH9 3FL, UK; 2 Department of Computer Science, University of Auckland, 303/38 Princes St, Auckland, 1010, NZ; 3 Centre for Computational Evolution, University of Auckland, 303/38 Princes St, Auckland, 1010, NZ; 4 Department of Microbiology and Immunology, Rega Institute, KU Leuven - University of Leuven, Herestraat 49, 3000 Leuven, Belgium; 5 Department of Human Genetics, University of California, Los Angeles, 695 Charles E. Young Dr., Los Angeles, CA 90095, USA; 6 Department of Biostatistics, University of California, Los Angeles, 650 Charles E. Young Dr., Los Angeles, CA 90095, USA

**Keywords:** Bayesian inference, Markov chain Monte Carlo, phylogenetics, visualization

## Abstract

Bayesian inference of phylogeny using Markov chain Monte Carlo (MCMC) plays a central role in understanding evolutionary history from molecular sequence data. Visualizing and analyzing the MCMC-generated samples from the posterior distribution is a key step in any non-trivial Bayesian inference. We present the software package *Tracer* (version 1.7) for visualizing and analyzing the MCMC trace files generated through Bayesian phylogenetic inference. *Tracer* provides kernel density estimation, multivariate visualization, demographic trajectory reconstruction, conditional posterior distribution summary, and more. *Tracer* is open-source and available at http://beast.community/tracer.

Bayesian inference of phylogeny using Markov chain Monte Carlo (MCMC) (Rannala and Yang 1996; Mau et al. 1999; Drummond et al. 2002) flourishes as a popular approach to uncover the evolutionary relationships among taxa, such as genes, genomes, individuals, or species. MCMC approaches generate samples of model parameter values—including the phylogenetic tree—drawn from their posterior distribution given molecular sequence data and a selection of evolutionary models. Visualizing, tabulating, and marginalizing these samples are critical for approximating the posterior quantities of interest that one reports as the outcome of a Bayesian phylogenetic analysis. To facilitate this task, we have developed the *Tracer* (version 1.7) software package to process MCMC trace files containing parameter samples and to interactively explore the high-dimensional posterior distribution. *Tracer* works automatically with sample output from BEAST (Drummond et al. 2012), BEAST2 (Bouckaert et al. 2014), LAMARC (Kuhner 2006), Migrate (Beerli 2006), MrBayes (Ronquist et al. 2012), RevBayes (Höhna et al. 2016), and possibly other MCMC programs from other domains.

## Design and Implementation

**Figure 1.**
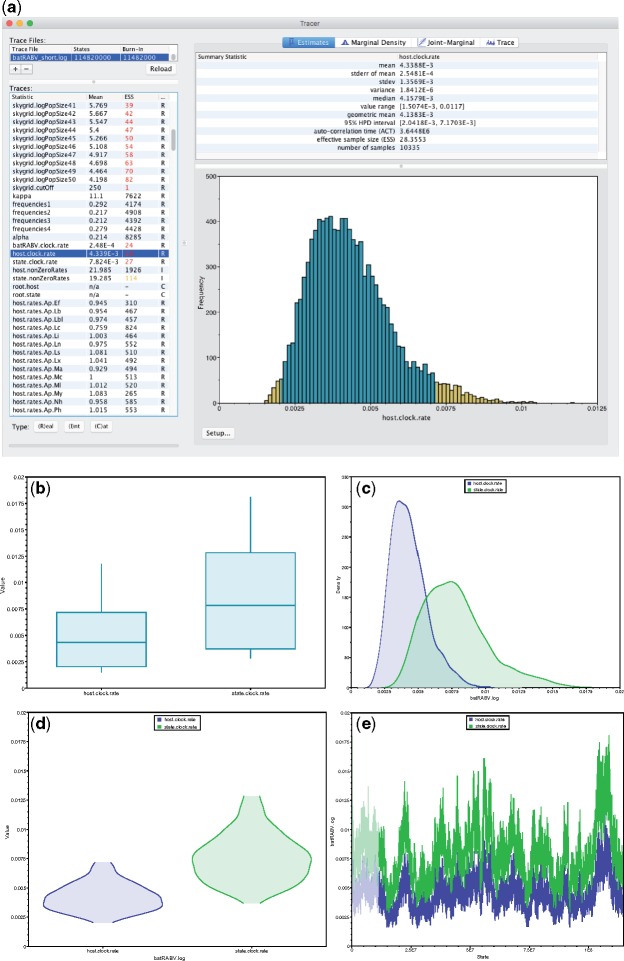
Overview of *Tracer* functionality and individual parameter visualizations: a) Main *Tracer* panel upon loading a single trace file; b) boxplot representation of two continuous parameters; corresponding c) kernel density estimates; d) violin plots; e) the actual traces connecting the parameter values visited by the Markov chain.

*Tracer* examines the posterior samples from all the available parameters—treating continuous, integer and categorical parameters appropriately—from a trace and presents statistical summaries and visualizations. Further, *Tracer* can analyze a single trace or combine samples from multiple files. Immediately apparent in the default *Tracer* view, the effective sample size (ESS) is one such statistic that allows users to assess the number of effectively independent draws from the posterior distribution the trace represents (Figure 1a). Color coding assists the user in determining potential MCMC mixing problems, with arbitrary cut-off values at 100 and 200.

Selecting multiple parameters from the “Traces” panel on the left generates a side-by-side comparison or an overlay of the selected parameters’ visualizations (Figure 1 b–e). Multiple trace files can be selected in a similar fashion to compare posterior samples between different replicates of an analysis. If multiple trace files contain the same collection of parameters, then a “Combined” trace appears automatically. *Tracer* generates four display panels for the selected parameters:

- Estimates: Reports common summary statistics such as the sample mean, standard deviation, highest posterior density interval, and ESS. Also presents a histogram of sample values for a single selected parameter (Figure 1a) or side-by-side boxplots for multiple continuous parameters (Figure 1b).
- Marginal density: Draws density plots for the selected parameter(s), including kernel density estimates (Figure 1c), histograms, and violin plots (Figure 1d) for continuous parameters and frequency plots for categorical or integer parameters.
- Joint-marginal: Visualization in this panel appears after selecting two or more parameters, and the plot form depends on the parameter types. We show several examples in the next section of the article.
- Trace: Constructs line plots connecting the sequential samples of one or more selected parameters against state or generation number (Figure 1e). Users typically use this plot to assess mixing, select a suitable burn-in and identify trends that suggest convergence issues.

*Tracer* offers a solution of visualizing conditional posterior distributions as well. Selecting one continuous and one integer or categorical parameter generates side-by-side violin or boxplots under the Joint-Marginal panel. These plots present the continuous parameter distribution conditioned on the unique integer or categorical values. A typical use case involves Bayesian stochastic search variable selection (BSSVS), a form of model averaging, in which parameters influence the likelihood function only when a specific model is selected by a random indicator function. Under BSSVS, a posterior estimate of the parameter should only sample values from states where the indicator equals one. Discrete phylogeographic analyses frequently employ BSSVS due to the potentially large amount of transition rates that need to be estimated (Lemey et al. 2009), but this is also relevant when employing model averaging approaches, e.g., over relaxed molecular clocks (Li and Drummond 2012).

Finally, *Tracer* provides demographic reconstruction resulting in a graphical plot, often applied to reconstruct epidemic dynamics. Available models include, e.g., constant size, exponential and logistic growth (Drummond et al. 2002), and the non-parametric Bayesian skyline (Drummond et al. 2005; Heled and Drummond 2008), skyride (Minin et al. 2008), and skygrid (Gill et al. 2013).

## Example

### Cross-Species Dynamics of North American Bat Rabies

We use *Tracer* to infer the spatial dispersal and cross-species dynamics of rabies virus (RABV) in North American bats. The data set comprises 372 *nucleoprotein* gene sequences from 17 bat species, sampled between 1997 and 2006 across 14 states in the United States (Streicker et al. 2010; Faria et al. 2013). We estimate RABV ancestral locations and host-jumping history using a Bayesian discrete phylogeographic approach with BSSVS, while simultaneously estimating effective population sizes over time through a Bayesian skygrid coalescent model (Gill et al. 2013).

**Figure 2.**
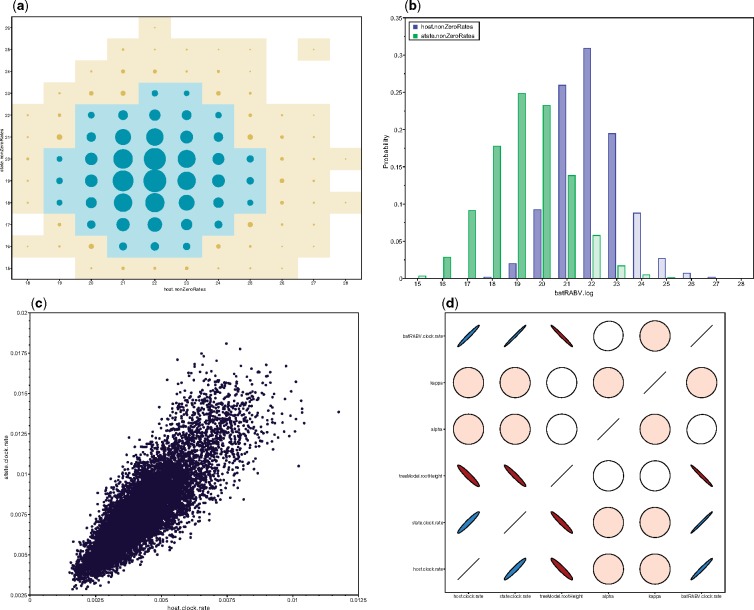
Multi-parameter visualizations of: a) the joint probability distribution of two integer variables through a bubble chart; b) the marginal density of multiple integer or categorical variables through frequency plots; c) two continuous variables through a classic scatter or correlation plot; d) multiple ($>2$) continuous variables using large correlation matrices.

Phylogeographic BSSVS inference includes parameters of both integer (number of non-zero transition rates) and categorical (host or location-state) trace types. In *Tracer*, a bubble chart visualizes the joint probability distribution between two integer or categorical traces (see Figure 2a). Circle area is proportional to the joint probability, with a colored tile background if this probability reaches a nominal threshold to enhance visibility. Marginal density plots can also display multiple integer parameters, each with unique colour scales (see Figure 2b). With approximately equal numbers of transition rates, both figures suggest similar host and location trait model complexity. *Tracer* also provides popular visualizations for continuous parameters, including scatter plots for two parameters (see Figure 2c), and extensions for correlations between $\ge 2$ continuous parameters (Figure 2d; Murdoch and Chow 1996). Colour gradients indicate strength and direction of the correlation, from red (strong negative) to blue (strong positive). Ellipse shapes re-enforce the strength of correlation, with no correlation appearing as a circle and perfect (anti)correlation as a line.

**Figure 3.**
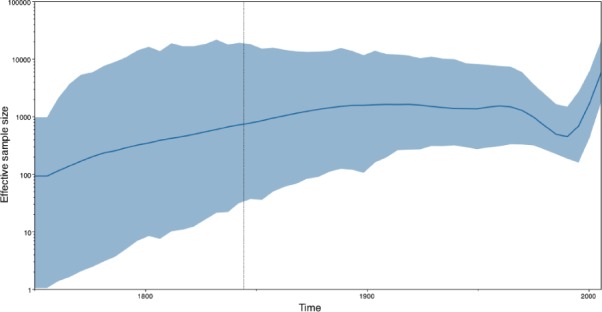
Estimating the effective population sizes over time using a Bayesian skygrid demographic reconstruction for rabies virus in North America.

*Tracer* reconstructs the demographic history of RABV by drawing the effective population sizes over time (Figure 3). RABV has successfully established itself in North American bat species, with its effective population size rising steadily throughout recent centuries. Following a rapid decline at the end of last century, we observe a recent sharp increase in size.

Other packages are available for the post-processing of MCMC samples. “coda” (Plummer et al. 2006) provides some of the functionality of *Tracer* within the R programming environment, while “AWTY” (Nylander et al. 2007) and “RWTY” (Warren et al. 2017) explore the convergence of the phylogenetic tree parameter itself across multiple MCMC runs. These alternative packages compute, e.g., Gelman–Rubin diagnostics (Gelman and Rubin 1992) that *Tracer* currently does not provide.

## Availability

*Tracer* is open-source under the GNU lesser general public license and available in both source code (https://github.com/beast-dev/tracer) and executable (http://beast.community/tracer) forms. This latter page also serves up self-contained, step-by-step tutorials covering basic to advanced usage of *Tracer* to summarize posteriors under a variety of phylogenetic models using BEAST and diagnose MCMC chain convergence. Popular tutorials employ *Tracer* to generate marginal parameter summaries and to infer population dynamics trajectories over time. *Tracer* requires Java version 1.6 or greater.
